# Incidence and severity of COVID‐19 in adults with and without HIV diagnosis

**DOI:** 10.1111/joim.13481

**Published:** 2022-03-21

**Authors:** Pontus Hedberg, Jan Vesterbacka, Ola Blennow, Catharina Missailidis, Piotr Nowak, Pontus Naucler

**Affiliations:** ^1^ Division of Infectious Diseases Department of Medicine Solna Karolinska Institutet Stockholm Sweden; ^2^ Department of Infectious Diseases Karolinska University Hospital Stockholm Sweden; ^3^ Department of Medicine Huddinge Infectious Diseases Karolinska Institutet Stockholm Sweden; ^4^ Department of Infectious Diseases/Venhälsan South General Hospital Stockholm Sweden

Dear Editor,

Previous reports have indicated that HIV infection is associated with worse COVID‐19 related outcomes [1, 2, 3] and that certain subpopulations among persons living with HIV (PLH) might be at a higher risk of severe COVID‐19 [4]. In this register‐ and population‐based cohort study, we analyzed the incidence and severity of COVID‐19 from 1 March 2020 to 31 August 2021 in individuals >18 years living with and without HIV in Stockholm, Sweden. Furthermore, we compared demographics, comorbidities, and characteristics of HIV infection in PLH not hospitalized and hospitalized with COVID‐19. Cohort definitions, data sources, study variables, and statistical analyses are presented in the supplement.

Out of 1,728,069 individuals ≥18 years in Region Stockholm, 3209 were PLH. Demographics and comorbidities of the general population and PLH are presented in Table S2. Among individuals living with and without HIV, the median age was 51 (interquartile range [IQR] 43–59) and 47 (IQR 33–63) years, and 51% and 42% had any risk factor for severe COVID‐19, respectively. The cumulative incidence of COVID‐19 by 31 August 2021 was 12% for both individuals living with and without HIV (Fig. S1).

Among 207,646 individuals with COVID‐19 during the study period, 364 (0.2%) were PLH. For each PLH, up to 10 individuals without diagnosed HIV were randomly selected, matching on the month of positive SARS‐CoV‐2 test, sex, age, and the number of comorbidity risk factors for severe COVID‐19. Baseline characteristics were balanced between PLH and the 3587 matched controls, except chronic kidney disease (PLH 4%, controls 2%, standardized mean difference [SMD] = 0.13) and substance use disorders (PLH 10%, controls 4%, SMD = 0.26) being more common in PLH and asthma being more common in controls (PLH 5%, controls 7%, SMD = 0.11) (Table 1). Among PLH and controls, 9% and 10% were hospitalized with COVID‐19 without intensive care unit (ICU) treatment, and 3% and 2% were hospitalized with COVID‐19 with ICU treatment, respectively. The 30‐day all‐cause mortality was 2% for PLH and 1% for controls. Compared to controls, the adjusted odds ratios (aOR) for COVID‐19 hospitalization without ICU treatment, with ICU treatment, and 30‐day all‐cause mortality in PLH were 0.97 (95% confidence interval (CI) 0.67–1.38), 1.51 (95% CI 0.68–3.14), and 1.26 (95% CI 0.45–3.03), respectively.

**Table 1:** Baseline characteristics and characteristics of COVID‐19 in the entire cohort, individuals with HIV, and matched controls

	Entire cohort	PLH	Controls	
	(*n* = 207,643)	(*n* = 364)	(*n* = 3587)	SMD
Baseline characteristics				
Male sex	99,260 (48)	250 (69)	2454 (68)	0.006
Age, years median (IQR)	44 (32–55)	50 (42–58)	50 (41–58)	0.044
18–29	42,298 (20)	17 (5)	170 (5)	
30–39	43,715 (21)	58 (16)	575 (16)	
40–49	44,993 (22)	102 (28)	996 (28)	
50–59	38,567 (19)	110 (30)	1089 (30)	0.008
60–69	19,281 (9)	62 (17)	607 (17)	
70–79	9427 (5)	12 (3)	120 (3)	
≥80	9362 (5)	3 (1)	30 (1)	
Living in nursing home	4896 (2)	3 (1)	24 (1)	0.018
No. of risk factors for severe COVID‐19^a^				0.034
0	123,311 (59)	175 (48)	1750 (49)	
1	51,124 (25)	99 (27)	988 (28)	
2	18,057 (9)	47 (13)	448 (13)	
3	8048 (4)	23 (6)	226 (6)	
4	4045 (2)	7 (2)	65 (2)	
>4	3058 (2)	13 (4)	110 (3)	
Asthma	11,799 (6)	17 (5)	261 (7)	0.110
Cancer	7761 (4)	19 (5)	210 (6)	0.028
Cerebrovascular disease	3493 (2)	10 (3)	82 (2)	0.029
Chronic kidney disease	3446 (2)	16 (4)	75 (2)	0.130
Chronic liver disease	916 (0)	6 (2)	37 (1)	0.054
Chronic lung disease	4077 (2)	9 (3)	96 (3)	0.013
Diabetes (type 1 or 2)	11,671 (6)	44 (12)	383 (11)	0.044
Heart disease	13,266 (6)	28 (8)	323 (9)	0.047
Hypertension	29,765 (14)	73 (20)	819 (23)	0.068
Immune deficiency	2210 (1)	7 (2)	71 (2)	0.004
Mental health disorders	40,439 (20)	80 (22)	730 (20)	0.040
Neurological disease, including dementia	7118 (3)	6 (2)	104 (3)	0.084
Substance‐use disorder	4128 (2)	38 (10)	135 (4)	0.262
Use of corticosteroids or other immunosuppressive drugs	4436 (2)	13 (4)	105 (3)	0.036
COVID‐19 characteristics				
Time period, positive test				–
Before June 2020	12,018 (6)	41 (11)	389 (11)	
June to September 2020	10,693 (5)	18 (5)	165 (5)	
October 2020 to February 2021	105,930 (51)	176 (48)	1755 (49)	
March to August 2021	79,002 (38)	129 (35)	1278 (36)	
Severity of COVID‐19				
Not hospitalized	191,335 (92)	321 (88)	3162 (88)	–
Hospitalized without ICU treatment	14,523 (7)	33 (9)	355 (10)	–
Hospitalized with ICU treatment	1785 (1)	10 (3)	70 (2)	–
Length of COVID‐19 hospitalization	7 (3–14)	6 (3–11)	7 (3–13)	–
30‐day all‐cause mortality	4162 (2)	6 (2)	44 (1)	–

*Note*: PLH was, for this analysis, defined as individuals having a B20‐B24 ICD‐10 diagnosis registered any time up to the day of first positive SARS‐CoV‐2 test. Individuals having their first HIV diagnosis registered after the COVID‐19 were excluded from this analysis (*n* = 3). Standardized mean differences (SMD) were used to assess cohort balance between PLH and matched controls. Continuous variables are presented as median (interquartile range) and categorical variables are presented as numbers (percentage).

Abbreviations: COVID‐19, coronavirus disease 2019; ICU, intensive care unit; IQR, interquartile range; PLH, people living with HIV; SMD, standardized mean differences.

^a^ The risk factors for severe COVID‐19 were based on the Centers for Disease Control and Prevention description of medical conditions associated with higher risk for severe COVID‐19; see Table S1 for a description of each condition.

Among PLH with COVID‐19, a total of 43 persons were hospitalized and 321 not hospitalized, with median ages of 59 years (IQR 55–67) and 48 years (IQR 41–56), respectively (Table S3). Among hospitalized and not hospitalized individuals, 16% and 2% had >4 risk factors for severe COVID‐19, respectively. Older age (50–64 and ≥65 years) was associated with hospitalization for COVID‐19, compared to <50 years of age, aOR 4.76 (95% CI 1.93–13.58) and 9.76 (95% CI 3.28–31.87), respectively. Time since HIV diagnosis and initiation of antiretroviral treatment were similar among both groups, whereas hospitalized individuals more often used protease inhibitors (Table S4). The median CD4 count was 630 (IQR 480–800) in not hospitalized individuals and 510 (IQR 390–690) in hospitalized individuals (*p*‐value = 0.04), whereas 6% and 8% had a detectable viral load. Twenty‐three percent of hospitalized PLH were treated in the ICU, and the 30‐day mortality was 5% compared to 1% among individuals not hospitalized with COVID‐19.

In this study, the cumulative incidence of COVID‐19 was similar in PLH and the general population. This observation is in line with previous studies from high‐income settings, but substantial differences between populations with different demographic and socioeconomic distributions might occur [5]. HIV infection was not associated with COVID‐19 hospitalization, and a relevant association between HIV infection and ICU admission or 30‐day all‐cause mortality could not be demonstrated nor excluded. Previous reports on the risk of severe outcomes in COVID‐19 in PLH are conflicting, with different study designs and comparison groups precluding direct comparisons [6]. Earlier studies have reported that older and frailer PLH with lower CD4+ counts and higher levels of HIV‐viremia have a higher risk of severe COVID‐19 [1, 7]. Herein, among PLH with COVID‐19, hospitalized patients were older, had more comorbidities, lower CD4+ counts, and more often were on protease inhibitors. The overrepresentation of protease inhibitor treatment in our hospitalized individuals may be a reflection of the use of these drugs being more common in older PLH.

Limitations of the study include limited sample size, and lack of body mass index and COVID‐19 vaccination data. A major strength of our study is the access to population‐based data on comorbidities, drug prescriptions, and SARS‐CoV‐2 positive tests, facilitating the matching of PLH with controls.

In summary, our study indicates that in a cohort of well‐treated PLH, HIV infection is not a risk factor for severe outcomes in COVID‐19.

## Funding information

The work was supported by EuCARE and Region Stockholm. P. H. was supported by Karolinska Institutet (combined clinical studies and PhD training program). The funders had no role in the design and conduct of the study; collection, management, analysis, and interpretation of the data; preparation, review, or approval of the manuscript; and decision to submit the manuscript for publication.

## Ethics approval and consent to participate

The need for consent was waived by the Swedish Ethical Review Authority (Dnr 2018/1030‐31, COVID‐19 research amendment Dnr 2020‐01385) since analyses are based on retrospectively collected data from the administrative health registry.

## Conflict of interests

None of the authors reports competing interests.

## Authors contributions

Pontus Naucler had full access to all the data in the study and takes responsibility for the integrity of the data and the accuracy of the data analysis. All authors have read and approved the manuscript.

Study concept and design: All authors Acquisition, analysis, or interpretation of data: PH, JV, PNo, Pna Drafting of the manuscript: PH, JV, CM, PNo, Pna Critical revision of the manuscript for important intellectual content: All authors Statistical analysis: PH.

## Supporting information


**Supplementary Material**. Cohort definitions, data sources, study variables, and statistical analyses.

## Data Availability

No data are available. Data from deidentified the administrative health registry are not freely available due to the protection of the personal integrity of the participants.
