# Supplementary material for: Incidence and severity of COVID‐19 in adults with and without HIV diagnosis
Source: J Intern Med. 2022 Mar 21;292(1):168–71. doi: 10.1111/joim.13481 (PMC9115199; doi:10.1111/joim.13481)
Supplement: Supplementary file 1 — Supplementary Material. Cohort definitions, data sources, study variables, and statistical analyses. [file JOIM-292-168-s001.docx]

Incidence and severity of COVID-19 in adults with and without HIV diagnosis

Authors: Pontus Hedberg, Jan Vesterbacka, Ola Blennow, Catharina Missailidis, Piotr Nowak, Pontus Naucler

Correspondending author: [pontus.hedberg@ki.se](mailto:pontus.hedberg@ki.se)

**Supplementary material**

Table of Contents

[Supplementary methods and materials 2](#_Toc96206931)

[Table S1. Definitions of study variables 4](#_Toc96206932)

[Table S2. Baseline characteristics in people living with HIV (PLH) and people not living with HIV 7](#_Toc96206933)

[Figure S1. Cumulative incidence of COVID-19 by 31 August 2021, in individuals living with (red line) and without (blue line) a HIV diagnosis in Stockholm, Sweden. 9](#_Toc96206934)

[Table S3. Baseline characteristics in people living with HIV (PLH), stratified by hospitalization for COVID-19 10](#_Toc96206935)

[Table S4. Characteristics of HIV-infection in people living with HIV (PLH), stratified by hospitalization for COVID-19 12](#_Toc96206936)

# Supplementary methods and materials

The Region Stockholm COVID-19 Cohort Study is a register-based observational population-based study of all individuals living in the Stockholm Region (catchment area approximately 2.4 million people). Within this study, we compared the cumulative incidence and severity of COVID-19 in adult individuals (>18 years) living with and without HIV. The cohort was restricted to individuals living in the Stockholm Region since at least one year before the start of the pandemic in order to be able to assess their previous medical history. Individuals were followed up for COVID-19 until 31 August 2021, moving out or death, whichever occurred first.

Data on demographics, medical diagnoses, outpatient and inpatient visits and drug prescriptions starting from 2010 was collected from an administrative health register (VAL; Stockholm regional healthcare data warehouse). Data on ICU treatment was available from a data extraction from the Swedish Intensive Care Registry. Data on positive SARS-CoV-2 PCR tests were obtained from a data extraction from the Public Health Agency of Sweden database SmiNet. Data on CD4 counts, CD4/CD8 ratios, and HIV-RNA were obtained from an EHR database extraction containing high-resolution clinical data since 2015 for patients with COVID-19. The study was approved by the Swedish Ethical Review Authority (Dnr 2018/1030-31, COVID-19 research amendment Dnr 2020-01385).

Definitions of study variables are presented in table S1. For the cumulative incidence analysis of COVID-19, HIV was defined as having an inpatient or outpatient HIV ICD-10 code (B20-B24) registered before the start of the pandemic. Individuals with a first diagnosis of HIV during the pandemic were excluded. The severity of COVID-19 was compared between PLH and up to 10 individuals living without diagnosed HIV using a matched cohort design. Matching was performed on month of positive SARS-CoV-2 test, sex, age category (18-29, 30-39, 40-49, 50-59, 60-69, 70-79, >80), and number of comorbidity risk factors for severe COVID-19 (0, 1, 2, 3, 4, >4)[4]. The severity of infection was categorized into not hospitalized, hospitalized without ICU treatment, hospitalized with ICU treatment within 14 days after the positive SARS-CoV-2 test and 30-day all-cause mortality after the first positive test.

Standardized mean differences (SMD) were used to assess cohort balance between PLH and matched controls. Baseline characteristics and characteristics of HIV infection in PLH not hospitalized and hospitalized with COVID-19 were analyzed with significance testing, with Chi squared test for categorical variables and t-test for continuous variables. A logistic regression model adjusting for age, sex and number of comorbidity risk factors for severe COVID-19 was used to compare COVID-19 hospitalizations and 30-day all-cause mortality among PLH and their matched controls. Among PLH, demographics, comorbidities and HIV-related characteristics, treatments and complications were compared between individuals not hospitalized and hospitalized with COVID-19, respectively. The groups were compared with a logistic regression model adjusted for age category (<50, 50-64, >65), sex and number of comorbidity risk factors for severe COVID-19 (0, 1, >2) adjusted logistic regression.

Statistical analyses were performed in R version 4.1.0.

#

# Table S1. Definitions of study variables

| **Variable** | **Definition** |
| --- | --- |
| **Study cohorts** |  |
| Fixed population cohort for analysis of cumulative incidence of COVID-19 | All individuals ≥ 18 years living in Stockholm Region for at least one year at the start of the COVID-19 pandemic (1 March 2020). Exclusion of individuals diagnosed with HIV after the start of the pandemic (n=85) for the analysis of COVID-19 incidence. |
| SARS-CoV-2 positive cohort | All individuals ≥ 18 years living in Stockholm Region for at least one year at the start of the COVID-19 pandemic (1 March 2020) with a positive test before 2021-09-01 to allow for assessment of severity of infection and 30-day all-cause mortality (end of study observation 2021-09-30). In this cohort, individuals diagnosed with HIV after the start of the pandemic could be excluded but were excluded if diagnosed with HIV after the first positive SARS-CoV-2 test (n=3). |
| **Assessment windows:** |  |
| Comorbidity assessment window (fixed population cohort) | From 2017-02-28 to 2020-02-29 |
| Comorbidity assessment window (SARS-CoV-2 positive cohort) | Three years to 30 days before the first positive SARS-CoV-2 test |
| Start of pandemic | 2020-03-01 |
| COVID-19 assessment window | 2020-03-01 – 2021-08-31 |
| End of study observation | 2021-09-30 |
| Censoring date | Date of death, moving out, or 2021-09-30, whichever comes first. |
| **Severity of COVID-19** |  |
| No COVID-19 hospitalization | No COVID-19 hospitalization recorded, see definition below. |
| COVID-19 hospitalization | A hospitalization with a U07.1 or U07.2 diagnosis at discharge, with the first positive SARS-CoV-2 test any time from 14 days before admission to the day of hospital discharge. |
| COVID-19 hospitalization with ICU-treatment | A COVID-19 hospitalization (see above) with an ICU admission any time from the day of hospital admission to hospital discharge, with a U07.1 or U07.2 diagnosis at discharge. |
| 30-day all-cause mortality | Mortality within 30 days after first positive SARS-CoV-2 test |
| **Comorbidity category (ICD-10)** |  |
| Asthma | J45 |
| Cancer | C00-C26, C30-C34, C37-C39, C40-C80, C81-C96 |
| Cerebrovascular disease | I60-I69 |
| Chronic kidney disease | N18 |
| Chronic liver diseases | B180E, B180G, B181E, B181G, B182E, B182G, B188E, B188G, B189E, B189G, K70, K71.7, K74, K75.4, K760 |
| Chronic lung diseases | I26, I27.0, I27.2, J44, J47, J70.2, J70.3, J70.4, J84, J98.2, J99.0, M05.1 |
| Diabetes mellitus (type 1 or 2) | E10-E14 |
| Heart disease | I05-I08, I20-I22, I24-I28, I34-I37, I42, I44-I50 |
| Hypertension | I10-I15 |
| Immune deficiencies | D70-D73.0, D80-D84, Z51.0, Z51.1 |
| Mental health disorders | F20-F29, F30-F39, F40-F48 |
| Neurologic conditions, including dementia | F01-F03, G10-G14, G20-G26, G30-G32, G70-G73, G80-G83. |
| Substance use disorder | F10-F19 |
| Use of corticosteroids or other immunosuppressive conditions | Two prescriptions of drug from ATC category H02 or L04 during the last year and one or more prescriptions during the last 90 days. |
| **Classification of antiretroviral drugs** | All antiretroviral drugs were based on one or more prescriptions during the last 180 days before the first positive SARS-CoV-2 test |
| Backbone antiretroviral nucleoside reverse transcriptase inhibitors, lamivudine/abacavir | J05AF05, J05AF06, J05AR02, J05AR13 |
| Backbone antiretroviral nucleoside reverse transcriptase inhibitors, tenofovir/emtricitabine | J05AR03, J05AR06, J05AR08, J05AR09, J05AR17, J05AR18, J05AR19, J05AR20 |
| Non-nucleoside reverse transcriptase inhibitors | J05AG01, J05AG03, J05AG04, J05AG05, J05AG06, J05AR08, J05AR11, J05AR19, J05AR21, J05AR24 |
| Protease inhibitors | J05AE08, J05AE10, J05AR10, J05AR14, J05AR22, J05AR26 |
| Integrase inhibitors | J05AJ01, J05AJ02, J05AJ03, J05AJ04, J05AR09, J05AR13, J05AR18, J05AR20, J05AR21, J05AR25, J05AX08, J05AX12 |
| Two drugs | Any of the following:   1. J05AR21 2. J05AR25 3. Integrase inhibitor + Protease inhibitor 4. Integrase inhibitor + non-nucleoside reverse transcriptase inhibitor 5. Non-nucleoside reverse transcriptase inhibitor + protease inhibitor |
| **HIV-related characteristics and complications** |  |
| CD4 count | The latest value taken from one year to one month before first SARS-CoV-2 positive test. |
| CD4/CD8-ratio | The latest value taken from one year to one month before first SARS-CoV-2 positive test. |
| HIV-RNA | The latest value taken from one year to one month before first SARS-CoV-2 positive test. |
| Sulfonamides and Trimethoprim treatment | Two or more prescriptions of J01EE during the last 180 days before first SARS-CoV-2 positive test. |
| Pneumocystosis or pneumocystis pneumonia | B20.6, B48.5, J17.2 |
| Tuberculosis | A15-A19 |
| Cytomegaloviral disease | B25 |
| Cryptococcosis | B45 |
| Toxoplasmosis | B58 |
| Lymphoma | C81-C86 |
| Kaposi sarcoma | B21.0, C46 |

**Abbreviations:**

ATC=Anatomical Therapeutic Chemical; COVID-19=Coronavirus disease 2019; HIV=Human Immunodeficiency Virus; ICD=International Classification of Diseases; ICU=Intensive Care Unit; SARS-CoV-2=Severe acute respiratory syndrome coronavirus 2

# Table S2. Baseline characteristics in people living with HIV (PLH) and people not living with HIV

|  | **PLH** **(n=3,209)** | **People not living with HIV** **(n=1,724,775)** |
| --- | --- | --- |
| Male sex | 2,154 (67) | 855,009 (50) |
| Age, years median (IQR) | 51 (43-59) | 47 (33-63) |
| 18-29 | 128 (4) | 304,281 (18) |
| 30-39 | 447 (14) | 322,923 (19) |
| 40-49 | 829 (26) | 298,420 (17) |
| 50-59 | 1,029 (32) | 289,546 (17) |
| 60-69 | 549 (17) | 218,659 (13) |
| 70-79 | 201 (6) | 188,447 (11) |
| >80 | 26 (1) | 102,499 (6) |
| Living in nursing home | 10 (0) | 15,356 (1) |
| No. risk factors for severe COVID-19 ^b^ |  |  |
| 0 | 1,560 (49) | 998,777 (58) |
| 1 | 874 (27) | 414,151 (24) |
| 2 | 443 (14) | 175,855 (10) |
| 3 | 191 (6) | 79,950 (5) |
| 4 | 75 (2) | 35,254 (2) |
| >4 | 66 (2) | 20,788 (1) |
| Asthma | 115 (4) | 85,357 (5) |
| Cancer | 159 (5) | 83,698 (5) |
| Cerebrovascular disease | 85 (3) | 28,935 (2) |
| Chronic kidney disease | 92 (3) | 26,824 (2) |
| Chronic liver disease | 57 (2) | 7,510 (0) |
| Chronic lung disease | 103 (3) | 42,145 (2) |
| Diabetes (type 1 or 2) | 296 (9) | 102,913 (6) |
| Heart disease | 206 (6) | 125,629 (7) |
| Hypertension | 672 (21) | 311,302 (18) |
| Immune deficiency | 46 (1) | 20,557 (1) |
| Mental health disorders | 707 (22) | 299,940 (17) |
| Neurological disease, including dementia | 76 (2) | 46,559 (3) |
| Substance use disorder | 325 (10) | 42,511 (3) |
| Use of corticosteroids or other immunosuppressive drugs | 59 (2) | 35,942 (2) |

a) Adjusted for age-category and sex.
b) The risk factors for severe COVID-19 were based on the Centers for Disease Control and Prevention description of medical conditions associated with higher risk for severe COVID-19, see table S1 for a description of each condition.  **Legend:** PLH was for this analysis defined as individuals having a B20-B24 ICD-10 diagnosis registered any time before 2020-03-01. Individuals having their first HIV diagnosis registered from the start of the pandemic and onwards were removed from this analysis (n=85). Continuous variables are presented as median (interquartile range), and categorical variables are presented as numbers (percentage).

**Abbreviations:** HIV=Human Immunodeficiency Virus; PLH=People living with HIV

# Figure S1. Cumulative incidence of COVID-19 by 31 August 2021, in individuals living with (red line) and without (blue line) a HIV diagnosis in Stockholm, Sweden.

**Abbreviations:** HIV=Human Immunodeficiency Virus; COVID-19=Coronavirus disease 2019

# Table S3. Baseline characteristics in people living with HIV (PLH), stratified by hospitalization for COVID-19

|  | **Not hospitalized (n=321)** | **Hospitalized (n=43)** | **P Value** |
| --- | --- | --- | --- |
| Male sex | 218 (68) | 32 (74) | 0.491 |
| Age, years median (IQR) | 48 (41-56) | 59 (55-67) | <0.001 |
| 18-29 | 17 (5) | 0 (0) |  |
| 30-39 | 57 (18) | 1 (2) |  |
| 40-49 | 97 (30) | 5 (12) |  |
| 50-59 | 93 (29) | 17 (40) | <0.001 |
| 60-69 | 47 (15) | 15 (35) |  |
| 70-79 | 7 (2) | 5 (12) |  |
| >80 | 3 (1) | 0 (0) |  |
| Living in nursing home | 3 (1) | 0 (0) | 1.000 |
| No. risk factors for severe COVID-19 ^a^ |  |  | <0.001 |
| 0 | 162 (51) | 13 (30) |  |
| 1 | 92 (29) | 7 (16) |  |
| 2 | 38 (12) | 9 (21) |  |
| 3 | 20 (6) | 3 (7) |  |
| 4 | 3 (1) | 4 (9) |  |
| >4 | 6 (2) | 7 (16) |  |
| Asthma | 11 (3) | 6 (14) | 0.007 |
| Cancer | 14 (4) | 5 (12) | 0.100 |
| Cerebrovascular disease | 8 (3) | 2 (5) | 0.752 |
| Chronic kidney disease | 10 (3) | 6 (14) | 0.004 |
| Chronic liver disease | 5 (2) | 1 (2) | 1.000 |
| Chronic lung disease | 5 (2) | 4 (9) | 0.011 |
| Diabetes (type 1 or 2) | 30 (9) | 14 (33) | <0.001 |
| Heart disease | 19 (6) | 9 (21) | 0.002 |
| Hypertension | 55 (17) | 18 (42) | <0.001 |
| Immune deficiency | 6 (2) | 1 (2) | 1.000 |
| Mental health disorders | 68 (21) | 12 (28) | 0.422 |
| Neurological disease, including dementia | 4 (1) | 2 (5) | 0.313 |
| Substance use disorder | 32 (10) | 6 (14) | 0.591 |
| Use of corticosteroids or other immunosuppressive drugs | 7 (2) | 6 (14) | 0.001 |
| Time period, positive test |  |  | <0.001 |
| Before June 2020 | 20 (6) | 21 (49) |  |
| June to September 2020 | 18 (6) | 0 (0) |  |
| October 2020 to February 2021 | 164 (51) | 12 (28) |  |
| March to August 2021 | 119 (37) | 10 (23) |  |

a) The risk factors for severe COVID-19 were based on the Centers for Disease Control and Prevention description of medical conditions associated with higher risk for severe COVID-19, see table S1 for a description of each condition.

**Legend:** PLH was for this analysis defined as individuals having a B20-B24 ICD-10 diagnosis registered any time up to the day of the first positive SARS-CoV-2 test. Individuals having their first HIV diagnosis registered after the COVID-19 were excluded from this analysis (n=3). Continuous variables are presented as median (interquartile range), and categorical variables are presented as numbers (percentage).

**Abbreviations:** COVID-19=Coronavirus disease 2019; HIV=Human Immunodeficiency Virus; IQR=Interquartile range

# Table S4. Characteristics of HIV-infection in people living with HIV (PLH), stratified by hospitalization for COVID-19

|  | **Not hospitalized (n=321)** | **Hospitalized (n=43)** | **P Value** |
| --- | --- | --- | --- |
| Time with HIV |  |  | 0.911 |
| <1 year | 7 (2) | 1 (2) |  |
| 1-5 years | 61 (19) | 7 (16) |  |
| >5 years | 253 (79) | 35 (81) |  |
| CD4 count, median (IQR)^a^ | 630 (480-800) | 510 (390-690) | 0.043 |
| <200 | 3 (1) | 2 (5) | 0.009 |
| 200-499 | 79 (27) | 18 (44) |  |
| >500 | 211 (72) | 21 (51) |  |
| CD4/CD8 ratio, median (IQR)^b^ | 0.92 (0.63-1.25) | 0.72 (0.46-1.27) | 0.113 |
| Plasma HIV RNA viral load^c^ |  |  | 0.984 |
| <50 copies/mL | 282 (94) | 37 (92) |  |
| >50 copies/mL | 18 (6) | 3 (8) |  |
| Time with antiretroviral treatment |  |  | 0.806 |
| No drug prescription registered | 17 (5) | 2 (5) |  |
| <1 year | 1 (0) | 0 (0) |  |
| 1-5 years | 54 (17) | 5 (12) |  |
| >5 years | 249 (78) | 36 (84) |  |
| Type of antiretroviral treatment |  |  |  |
| Backbone lamivudine/abacavir | 106 (33) | 14 (33) | 1.000 |
| Backbone tenofovir/emtricitabine | 165 (51) | 19 (44) | 0.468 |
| NNRTI | 84 (26) | 12 (28) | 0.953 |
| Protease inhibitors | 17 (5) | 7 (16) | 0.016 |
| Integrase inhibitors | 201 (63) | 27 (63) | 1.000 |
| Protease inhibitors and integrase inhibitors | 8 (3) | 4 (9) | 0.058 |
| Two drugs | 39 (12) | 9 (20) | 0.174 |
| HIV-related complications |  |  |  |
| Use of Sulfonamides and Trimethoprim | 0 (0) | 1 (2) | 0.236 |
| Pneumocystis infection | 0 (0) | 0 (0) | NA |
| Tuberculosis | 0 (0) | 0 (0) | NA |
| Cytomegaloviral disease | 0 (0) | 0 (0) | NA |
| Cryptococcosis | 0 (0) | 0 (0) | NA |
| Toxoplasmosis | 0 (0) | 0 (0) | NA |
| Lymphoma | 1 (0) | 0 (0) | 1.000 |
| Kaposi sarcoma | 0 (0) | 0 (0) | NA |

a) Data missing for 28 PLH not hospitalized and 2 PLH hospitalized with COVID-19.
b) Data missing for 28 PLH not hospitalized and 2 PLH hospitalized with COVID-19.
c) Data missing for 21 PLH not hospitalized and 3 PLH hospitalized with COVID-19.

**Legend:** PLH was for this analysis defined as individuals having a B20-B24 ICD-10 diagnosis registered any time up to the day of the first positive SARS-CoV-2 test. Individuals having their first HIV diagnosis registered after the COVID-19 were excluded from this analysis (n=3). Continuous variables are presented as median (interquartile range), and categorical variables are presented as numbers (percentage).

**Abbreviations:** COVID-19=Coronavirus disease 2019; HIV=Human Immunodeficiency Virus; IQR=Interquartile range; NNRTI= Non-Nucleoside Reverse Transcriptase Inhibitor
